# The role of faith in parenting; considerations when implementing family skills interventions with families affected by armed conflict or displacement

**DOI:** 10.3389/fpsyt.2023.1118662

**Published:** 2023-02-23

**Authors:** Aala El-Khani, Rachel Calam, Wadih Maalouf

**Affiliations:** ^1^United Nations Office on Drugs and Crime, Vienna, Austria; ^2^Division of Psychology and Mental Health, The University of Manchester, Manchester, United Kingdom

**Keywords:** religion, parenting, conflict, refugee, family skills

## Abstract

Religious beliefs and practices are fundamental to shaping family functioning in many countries and cultures around the world. They are often associated with a strong influence on parenting, and a potential resource for parents. While nurturing caregiving can act as a protective shield, buffering against the negative effects on children’s well-being, armed conflict and displacement often compromises parental well-being and positive parenting practices. Making interventions available to families affected by conflict and displacement that help to develop the quality of parenting is now seen as an important component in the care of war-affected children, causing a rise in family skills interventions for humanitarian contexts. Accordingly, there are certain considerations that need to be taken to achieve cultural sensitivity and acceptability, that account for the influence of religion. Here we share our United Nations Office on Drugs and Crime (UNODC) experience in the case of implementing “Strong Families,” a UNODC family skills programme implemented in over 30 countries, providing key recommendations. (1) Appreciate and account for common religious beliefs and practices in your target populations; (2) ensure programme material acceptability and sensitivity; (3) avoid initiation of direct discussions, on religious beliefs or practices; and (4) facilitator need to be trained and prepared to respond to questions about faith. Though these considerations are presented considering the implementation of family skills programmes, they are also relevant to a range of other programming in which direct social (or other) contact is made with families in challenged contexts, aiming to reduce any perceived gaps between trainers and the families they are working with, and give families a sense that their religious beliefs, values, and priorities are understood.

## The role of faith in parenting

Religious beliefs and practices are fundamental to shaping family functioning in many countries and cultures around the world, often associated with a strong influence on parenting. The literature is rich in studies that have shown a positive relationship between internal aspects related to faith, such as religious behavior, religious adherence, and a strong faith, and an individual’s level of resilience (1, 2). Research exploring links between religiosity (the degree and quality of being religious), and family well-being and outcomes, often stresses religion as a potential resource for parents (3, 4).

Parental religiosity, particularly in times of stress, is often associated with numerous positive family outcomes, including lower parental stress, increased parenting satisfaction and greater marital harmony (5, 6). Research exploring the reasons for these positive “side-effects” experienced by caregivers relate to parents sanctifying their roles by connecting parenting activities with sacred meaning. This can make challenges feel more tolerable, manageable, worthwhile, rewarding, and also increase adaptive functioning (6).

These positive effects of parental religiosity can benefit children too, with an association with parental responsiveness and higher levels of affection between children and their parents (5–8). A study with African Americans of low socioeconomic status indicated that religiosity and spirituality were positively correlated with fewer dysfunctional parent-child interactions (9). In a study with Christian mothers, the majority of mothers regularly engaged in bi-directional communication with their children, a component of parenting that has significant benefits to children later on in their life (10). Also, children who attend religious activities with their parent are more likely to experience higher levels of psychological well-being throughout adolescence (11), with parents’ religiosity related inversely to substance use and externalizing problems among adolescents (12–14). These positive effects may be due to religious norms emphasizing and encouraging the importance of families spending time together which allows for opportunities for caregivers to engage in high quality interactions with their children (15). Advantages have also been found for families that take part in certain group religious rituals, even when they might not practice religious adherence or other religious behaviors (16). One example is in a study of Jewish families’ celebration of the Sabbath (i.e., the lighting of the candles and sacred prayers and blessings), which was found to serve as a family strengthening practice and promoted a healthy child-adult relationship (17).

In contrast to positive relationships between concepts of parenting and both religiosity and belonging to a faith group, there is also research that identifies religion as one potential risk factor for child maltreatment. Several studies have confirmed a relationship between membership in a conservative religious organization and approval of, as well as more frequent use of, corporal punishment [e.g., (18)]. For example, in a qualitative study of Ultra-Orthodox religious communities, social workers noted that children who are brought up within may be exposed to risks such as a biblical view supporting corporal punishment (19). The use of referencing of religious texts as reasons for child maltreatment is not specific to any one religion, rather being seen across all major religious groups (20). Also, children and adolescents who go against the teachings of their religious community may find themselves shunned by their families and community (21).

## The role of families during war and displacement

In war and displacement contexts, family is often the only remaining institution in the lives of children. While nurturing caregiving can act as a protective shield, buffering against the negative effects on children’s well-being, armed conflict and displacement often compromises parental well-being and positive parenting practices. This can be a direct source of risk for children (22), with parents experiencing high stress less likely to provide children with the positive interactions that promote healthy psychosocial and physical development (23). Instead, children in such contexts are more likely to experience harsh parenting with increasing children’s risk of a variety of enduring emotional and behavioral problems (24).

Faith within the family was one aspect in the literature reflecting the positive role parenting can play in such circumstances. For instance, one study with Muslim Syrian refugee families residing in refugee camps revealed that despite the extremely challenging circumstances they had experienced and were still going through, parents maintained a positive outlook for their families and were motivated to improve their parenting as well as meet the new parenting challenges they faced. They referred to their faith often, indicating their faith as a motivator and believed God would reward their hardships (25). Another study with African refugees resettled in the USA found that they used spirituality to heal both the physical body and the mind from post-traumatic experiences. Religiosity was shown to be one of the highest coping mechanisms used by the African immigrants to overcome the many struggles they faced in their journey through displacement to resettlement (26). It is only in recent years that there has been a renewed interest in how faith can be a positive agent of change in humanitarian support (27), and that the role of the family can be central to family wellbeing (28).

Making interventions available to families affected by conflict and displacement that help to develop the quality of parenting is now seen as an important component in the response to the care of war-affected children (28). This has caused a rise in parenting and family skills interventions for humanitarian and low resource contexts (29). Family skills interventions offer a combination of parenting knowledge, skill building, competency enhancement and support (30). They aim to strengthen family protective factors such as communication, trust, problem-solving skills and conflict resolution, and strengthen the bonding and attachment between caregivers and children. All families in all contexts can greatly benefit from engaging with family skills resources.

## The Strong Families programme

For over a decade, the United Nations Office on Drugs and Crime (UNODC) has worked to provide a pyramid model of interventions aimed at fostering strong relationships within families, and good mental health for children as a preventive strategy against many negative social and health consequences. These draw from the best evidence available in the literature internationally, namely as guided by the UNODC WHO International Standards on Drug Use Prevention (31). This pyramid brought together brief resources and interventions to provide a stepped model for parenting through armed conflict and disaster.^1^ These resources have been developed for contexts where resources may be scarce. At the bottom of the model are brief, light touch self-read and self-help resources with universal parenting messages, such as videos, leaflets and booklets. The pyramid moves to more specialized interventions at the top of the model for families with a higher level of need, such as a modified trauma-based package for parents and their children (32). The pyramid intends to further expand to integrate further open-sourced packages developed through interagency efforts that further support parenting in such circumstances.

The first manualized parenting package that require a multi-session (yet light) mediation through a trained facilitator is UNODC Strong Families programme. The Strong Families programme is a selective, brief, family skills prevention intervention, designed to improve parenting skills, family resilience (33), as well as child well-being and family mental health. The Strong Families programme is aimed at families with children aged between eight and fifteen years living in stressful settings (including in challenged and humanitarian settings). The goal of the Strong Families programme is to support families in both recognizing their strengths and skills, and further building on their strengths by sharing their challenges, as well as the things that work for them. Over the past 5 years we have implemented the programme in over 25 countries with multiple studies conducted on the feasibility, acceptability, and effectiveness of the programme (33–35).

The extensive global implementation experience has provided the programme’s international trainers with key valuable insights on achieving cultural sensitivity and acceptability. In each country we further implement in, we reflect and adapt our training based on lessons learnt. We share these insights below.

## Considerations for training and implementation of family skills interventions

Religious beliefs and practices as such can be highly significant to shape caregiver behavior and family functioning. Moreover, such beliefs may be particularly valuable and sensitive in times of armed conflict or other situations of extreme stress. Accordingly, there are certain considerations that need to be taken especially when packages are mediated through a trained facilitator. Our experience in the case of implementation of UNODC Strong Families generated the below:

1. Appreciate and account for common religious beliefs and practices in your target populations (ideally work with trained facilitators from the same communities): Taking time to understand the common religious beliefs and practices of families is valuable to ensure better participation and engagement. The context of organized religion may function as a protective factor for families living in adverse circumstances, including displacement or low resource contexts. Thus, it is valuable for facilitators to appreciate the significance of the beliefs of the families that they are working with. The first step in preparation to work with any new population of families is to take time to engage with field staff to work to understand what, if any, cultural or religious beliefs the families adhere to and are common.

There can be significant conflict between religious theologies within and across religions, and religious practices across regions might differ, hence dwelling on the local contexts of such practices is valuable. The core of most religious traditions promote positively framed virtues and values of parenting in which children are treasured and parents hold expectations of caring and facilitative parenting for themselves and their children (4). Sometimes religious perception can greatly affect the engagement of participants in any family skills programme. For example, a common topic in the literature on religiosity of Muslims is the concept of “Sabr.” “Sabr” in Arabic translates directly as “to have patience,” and Muslims are encouraged by their faith traditions to hold virtues of patience when going through challenging times, and not be in the habit of excessive complaining, so as to receive divine blessings from God (36). For some Muslims, their interpretation of “sabr” might hinder their ability to engage in support and to not search for help, or not discuss their challenges with others, believing this may lessen their potential reward from God. But in parallel to “sabr,” the Muslim faith places strong emphasis, and even commands one to seek and use means of healing available to them in the world (37). In our implementation of Strong Families in communities with high numbers of Muslims, such as in Egypt, Palestine, and in Iran, we find that a facilitator working with Muslim families is far more likely to successfully manage conversations of differing opinions when they have first taken time to understand such key concepts in motivations and barriers to engagement in support. That is why having facilitators trained that are coming from similar cultural background of the target groups is ideal.

2. Ensure programme material acceptability and sensitivity: During the translation and material adaptation process, before launching a family skills package like Strong Families in any new country, a process of cultural adaptation with field staff is required. For example, the adaptation of the Strong Families programme to the Afghan context was assured through a working group with representatives from the Afghan Ministries of Public Health, Labour and Social Affairs, Counter Narcotics, Education and from Afghan non-governmental organizations and UNODC. In addition, three lead family skills trainers attended a 3-workshop discussing cultural appropriateness followed by local UNODC and Afghan delegation reviews inside the country. This adaptation in each country of implementation is further supported by a small-scale pilot of the materials where beneficiaries input is also accrued, this could include better tailoring specific messages including through faith-based information as necessary. The adaptation, analysis and review include content related adaptations, such as content examples, or images used in the materials, or the examples given in role play activities. This might also include adaptations beyond the training manual content and rather stretch to the process of its implementation, including how to recruit families, when to best approach them, when would it be best (day of the week and time of the day), when is ideal to run the package and beyond. Many of these process-related adaptations can be influenced by faith and religious practices.
3. Avoid initiation of direct discussions (or invitations to discussions), on religious beliefs or practices with families: This is done with the recognition that faith can be a highly personal and highly emotionally charged subject. Facilitators are also trained not to make any generalizations or hold expectations, no matter in which country or community the intervention is being implemented, on their personal belief of any programme participant’s practices (or lack of) of a certain faith. During the training of facilitators, role plays are conducted and written, and verbal scenarios explored to strengthen facilitators skills on this topic. Facilitators are also sensitized on the fact that just as religion can be a source of strength to families, it can also be a source of mental distress when families that have experienced challenges feel their beliefs have been shaken. It is not uncommon to find a parent who had a strong belief that God would protect them if they adhered to their religious practice, suddenly questioning their faith when they experience devastating pain, such as the loss of a child or a kidnaping of a partner. During debriefing sessions between trainers and facilitators implementing with families that have experienced significant trauma experiences, such as Afghan refugees in Serbia and with refugees in Cox Bazaar, experiences have been shared of caregivers questioning their faith. These facilitator experiences are always noted and then used as real examples in the following trainings in other countries.

While such knowledge of religious faith and practices is key in terms of implementation, it should remain as background knowledge facilitating and strengthening the messages and skills aimed to be developed during session. Moreover, as time of implementation of session is limited, facilitators face the challenging role of balancing time limitations with rolling out the entire content of the sessions and opening up such emotionally charged discussions can digress the focus and intent of the ongoing family session.

4. Facilitator need to be trained and prepared to respond to questions about faith: Just because such discussions should not be initiated, does not entail that the facilitators do not need to be trained on how to be prepared to address such discussions. We find families around the world naturally make parallels between the intervention and their faith beliefs and practices. For example, families learn two stress relief activities during the Strong Families intervention, one is “slow and gentle breathing” and the other “tapping” (also known as Emotional freedom technique, EFT). When implementing in counties such as India, Bangladesh and the Philippines, families often share that there is already significant use of holistic alternative medicine strategies in their faith practices, many of which stem from these two techniques, so they are familiar with such activities. This often leads families to want to share their beliefs and experiences. Thus, we place significant emphasis during facilitators’ training on how to respond to families’ comments, questions and group discussions concerning faith. This is achieved through a range of strategies, such as role play and discussions. Skills covered include managing conversations on differing group participants’ opinions and how to allow opportunities for caregivers to reflect on how their beliefs are affecting their decision-making process regarding parenting. Given diversity of backgrounds of families displaced during armed conflicts, facilitators may be faced with families expressing views that may be contentious and very different to their own personal views or of many other participating members of a parenting group. For example, families may have very varied beliefs on permissibility of their children dating and engaging in sexual behavior, experimenting with smoking or drugs or gender roles. It is important that the facilitator keeps in mind that their role is not to change beliefs or opinions, but rather to present opportunities for parents to learn how to improve their family functioning with evidence-based strategies that have been demonstrated to be successful with families. The trainer must maintain respect for the family members, and their values, at all times and not allow others to disregard or mock the opinions and thoughts a member may share with the trainer.

This extensive global experience cumulated through implementing Strong Families worldwide indicates that with skillful facilitation, parents often make links between their religious beliefs and programme components, reassuring themselves that what they are doing fits in with their values. Parents may find the connection they make between the programme and their beliefs valuable as they engage in and complete the programme.

Though these considerations are presented considering the implementation of family skills programmes, these considerations are also relevant to a range of other programming in which direct social (or other) contact is made with families in challenged and low resource contexts. Such considerations indicate a range of important ways in which programme trainers and facilitators can help to reduce any perceived gaps between themselves and the families they are working with, and give families a sense that their religious beliefs, values, and priorities are understood. They are relevant for families at whatever stage they are in their resettlement journey, and we recommend they are kept at the forefront of global programme implementation.

## Data availability statement

The original contributions presented in this study are included in the article/supplementary material, further inquiries can be directed to the corresponding author.

## Author contributions

All authors listed have made a substantial, direct, and intellectual contribution to the work, and approved it for publication.

## Footnotes

1
https://www.unodc.org/unodc/en/prevention/family-skills.html
